# Functional Properties of Dendritic Gap Junctions in Cerebellar Golgi Cells

**DOI:** 10.1016/j.neuron.2016.03.029

**Published:** 2016-06-01

**Authors:** Miklos Szoboszlay, Andrea Lőrincz, Frederic Lanore, Koen Vervaeke, R. Angus Silver, Zoltan Nusser

**Affiliations:** 1 Laboratory of Cellular Neurophysiology, Institute of Experimental Medicine of the Hungarian Academy of Sciences, Budapest 1083, Hungary; 2 János Szentágothai School of Neurosciences, Semmelweis University, Budapest 1085, Hungary; 3 Department of Neuroscience, Physiology, and Pharmacology, University College London, London WC1E 6BT, UK

**Keywords:** gap junctions, electrical synapses, dendrites, interneuron, cerebellum, immunolocalization, connexin36

## Abstract

The strength and variability of electrical synaptic connections between GABAergic interneurons are key determinants of spike synchrony within neuronal networks. However, little is known about how electrical coupling strength is determined due to the inaccessibility of gap junctions on the dendritic tree. We investigated the properties of gap junctions in cerebellar interneurons by combining paired somato-somatic and somato-dendritic recordings, anatomical reconstructions, immunohistochemistry, electron microscopy, and modeling. By fitting detailed compartmental models of Golgi cells to their somato-dendritic voltage responses, we determined their passive electrical properties and the mean gap junction conductance (0.9 nS). Connexin36 immunofluorescence and freeze-fracture replica immunogold labeling revealed a large variability in gap junction size and that only 18% of the 340 channels are open in each plaque. Our results establish that the number of gap junctions per connection is the main determinant of both the strength and variability in electrical coupling between Golgi cells.

## Highlights

• The mean conductance of dendritic gap junctions between Golgi cells is 0.9 nS
• Dendritic gap junctions have 340 connexin36 channels of which 18% are open
• Dendritic location contributes little to coupling strength variability
• Gap junction number is the main determinant of coupling strength variability

## Teaser

Variation in the strength of electrical synapses influences spike synchrony in interneuron networks. Szoboszlay et al. investigated the properties of electrical synapses between cerebellar Golgi cells and showed that the number of gap junctions is the main determinant of coupling strength variability.

## Introduction

Electrical synapses formed by gap junctions (GJs) mediate intercellular communication by allowing direct flow of current between neurons. GJ-mediated electrical coupling between inhibitory interneurons (INs) has been observed in many regions of the brain including the thalamus (Hughes et al., 2002, Landisman et al., 2002), hippocampus (Baude et al., 2007, Kosaka and Hama, 1985), neocortex (Galarreta and Hestrin, 1999, Gibson et al., 1999, Tamás et al., 2000), and cerebellar cortex (Alcami and Marty, 2013, Dugué et al., 2009, Mann-Metzer and Yarom, 1999, Sotelo and Llinás, 1972). It is well established that an action potential (AP) depolarizes its electrically coupled neighbors. The resulting GJ potential (GJP) or ‘spikelet’ entrains firing in the electrically coupled INs, enabling coordinated periodic chemical synaptic inhibition of the target principal cell populations. GJPs are thought to play a key role in generating network synchronization and oscillations (Beierlein et al., 2000, Deans et al., 2001, Dugué et al., 2009, Galarreta and Hestrin, 2001a, Hormuzdi et al., 2001, Hughes et al., 2002, Traub et al., 2001), with more strongly coupled networks tending to be more synchronized (Gibson et al., 2005, Hjorth et al., 2009, Kopell and Ermentrout, 2004, Traub et al., 2001). However, GJPs can also have a hyperpolarizing action: low pass filtering imposed by the dendrites and the GJs preferentially attenuates the rapid depolarizing component of the AP, while having a much smaller effect on the slow afterhyperpolarization component, which are large in many types of IN (Dugué et al., 2009, Galarreta and Hestrin, 2001b, Galarreta and Hestrin, 2002, Vervaeke et al., 2010). Our previous work has demonstrated that inhibitory GJPs can either synchronize or desynchronize cerebellar Golgi cell (GoC) networks, depending on the timing of synaptic excitation in relation to the phase of the ongoing oscillation (Vervaeke et al., 2010). Moreover, desynchronization of GoC networks critically depends on the heterogeneity in the strength of electrical coupling between GoCs (Vervaeke et al., 2010). However, the mechanisms underlying the strength and variability of electrical synapses formed between inhibitory INs are poorly understood.

Immunohistochemical and electron microscopic (EM) analysis have shown that GJs composed of connexin36 (Cx36) are predominantly located on the dendrites of inhibitory INs (Baude et al., 2007, Fukuda, 2009, Fukuda and Kosaka, 2000, Fukuda et al., 2006, Szabadics et al., 2001, Vervaeke et al., 2010). The dendritic location of GJs makes it difficult to determine their strength because the soma-to-soma attenuation of the membrane voltage reflects the combined effect of the dendrites and the GJs, leaving their individual contributions unknown. Moreover, much less is known about the passive and active properties of IN dendrites than for principal cells, due to the difficulty in making direct electrical recordings from their dendrites, which are often <1 μm in diameter. At chemical synaptic connections, how the number of postsynaptic receptors, their open probability, the number and dendritic location of synaptic contacts and dendritic filtering contribute to synaptic strength is reasonably well established, but these factors are largely unexplored for electrical synapses.

Here, we investigated the key factors that determine the strength of electrical synapses formed between cerebellar GoCs. We focused on quantifying the functional properties of GJs and determining the contribution of passive dendritic properties to the coupling strength. To address these, we performed dual somato-dendritic and paired somatic patch-clamp recordings in combination with light microscopic (LM) reconstructions and EM quantification of the number and location of GJs together with multi-compartmental modeling to reveal the conductance of GJs. By visualizing Cx36-containing connexons in identified GJs with SDS-digested freeze-fracture replica labeling (SDS-FRL), we have estimated the total number and open probability of channels within a dendritic GJ plaque.

## Results

### Number and Location of GJs in Electrically Coupled Cerebellar GoCs

**Figure 1:**
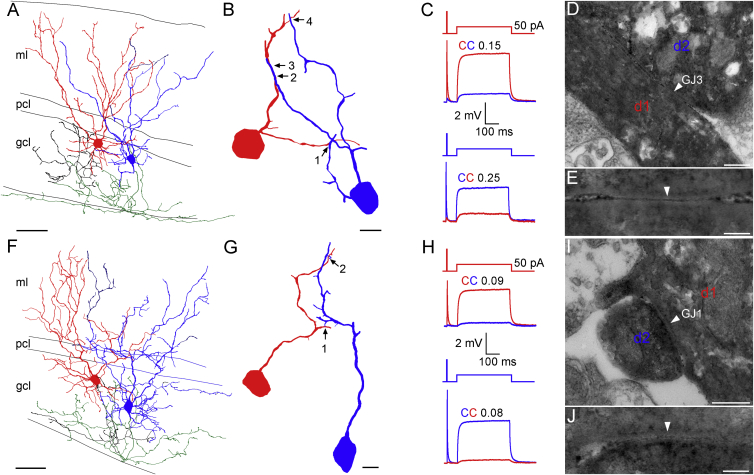
Determining the Strength of Electrical Coupling between GoCs and the Number and Location of GJs (A) LM reconstruction of two strongly coupled GoCs filled with biocytin (GoC1: soma and dendrites: red and axon: black and GoC2: soma and dendrites: blue and axon: green). (B) High-magnification view of EM-determined GJ locations (arrows) of the GoC pair in (A). The non-connecting dendrites and axons are not shown. (C) Voltage responses recorded in the GoC pair shown in (A) in response to current injections (2 ms 200 pA and 400 ms 50 pA). (D) EM identification of a GJ (GJ3; arrowhead) formed by dendrites d1 and d2 of the cells shown in (B). (E) High-magnification image of the GJ3 shown in (D). (F) As in (A), but for a weakly coupled GoC pair. (G) As in (B), but for a weakly coupled GoC pair. (H) As in (C), but for a weakly coupled GoC pair. (I) EM identification of a GJ (GJ1; arrowhead) formed by dendrites d1 and d2 of the cells shown in (G). (J) High-magnification image of GJ1 shown in (I) (Abbreviations: molecular layer, ml; Purkinje cell layer, pcl; and granule cell layer, gcl). The scale bars represent 50 μm in (A) and (F); 10 μm in (B) and (G); 200 nm in (D) and (I); and 50 nm in (E) and (J).

The coupling strength of electrical connections between cerebellar GoCs is highly variable and decreases as a function of distance between the two coupled GoC somata (Dugué et al., 2009, Vervaeke et al., 2010). However, prominent heterogeneity in coupling strength is also present among GoCs having similar intersomatic distances (Vervaeke et al., 2010). To investigate the relationship between the coupling strength and the number and location of GJs, we performed whole-cell current-clamp recordings from electrically coupled GoC pairs in acute slices obtained from the cerebellar vermis of young-adult mice. Electrical coupling strength was quantified using the coupling coefficient (CC), which is the ratio of the postsynaptic and presynaptic voltage changes during long current injections (400 ms and 50 pA) into the presynaptic GoC. Since active conductances in the perisomatic region (Vervaeke et al., 2012) can introduce a voltage dependence to the CC (Dugué et al., 2009, Haas et al., 2011), we made recordings in the presence of a cocktail of antagonists and channel blockers, that rendered the cell membranes passive (Experimental Procedures). Figure 1 shows two GoC pairs with similar soma-soma distances (57 μm and 70 μm), but with different CCs (Figures 1C and 1H). Both pairs exhibited asymmetry in CCs with CC_12_ = 0.15 and CC_21_ = 0.25 for the first (Figure 1C) and CC_12_ = 0.09 and CC_21_ = 0.08 for the second pair (Figure 1H). Across all paired recordings, the ratio of larger to smaller CCs (1.31 ± 0.05, n = 21 pairs) was similar to the ratio of the input resistances (R_in_) of the coupled GoCs (1.26 ± 0.04, n = 21 pairs and p = 0.16, paired t test), suggesting the soma-to-soma conductance of the connection was symmetrical (see later for quantification of GJ plaque conductance: G_GJ_). Following the recordings, the cells were fixed and processed for correlated LM and EM analysis. In four pairs, we were able to perform a full anatomical analysis, which revealed variable numbers of GJs (range: 1–4, n = 4 pairs) and GJ locations. In total, nine GJs were identified in four GoC pairs, one of which connected basal dendrites and eight GJs were located on apical dendrites. The GoC pair with a high CC shown in Figure 1A was connected by four GJs (Figures 1A–1E), whereas the more weakly coupled pair had only two GJs (Figures 1F–1J). Analysis of two other pairs revealed a strongly coupled pair (soma-soma distance 56 μm; CC_12_ = 0.2 and CC_21_ = 0.16) connected by two GJs and a weakly coupled pair (soma-soma distance 39 μm; CC_12_ = 0.11 and CC_21_ = 0.1) with only one GJ. The strongest connection (CC_21_ = 0.25 and CC_12_ = 0.15) was mediated by proximal GJs (mean distances from soma: 45 and 62 μm), whereas the weakest connection (CC_21_ = 0.08 and CC_12_ = 0.09) was mediated by more distal GJs (95 and 128 μm).

### The Strength of Cx36 Puncta in GoCs and Their Dependence on Distance from Soma

**Figure 2:**
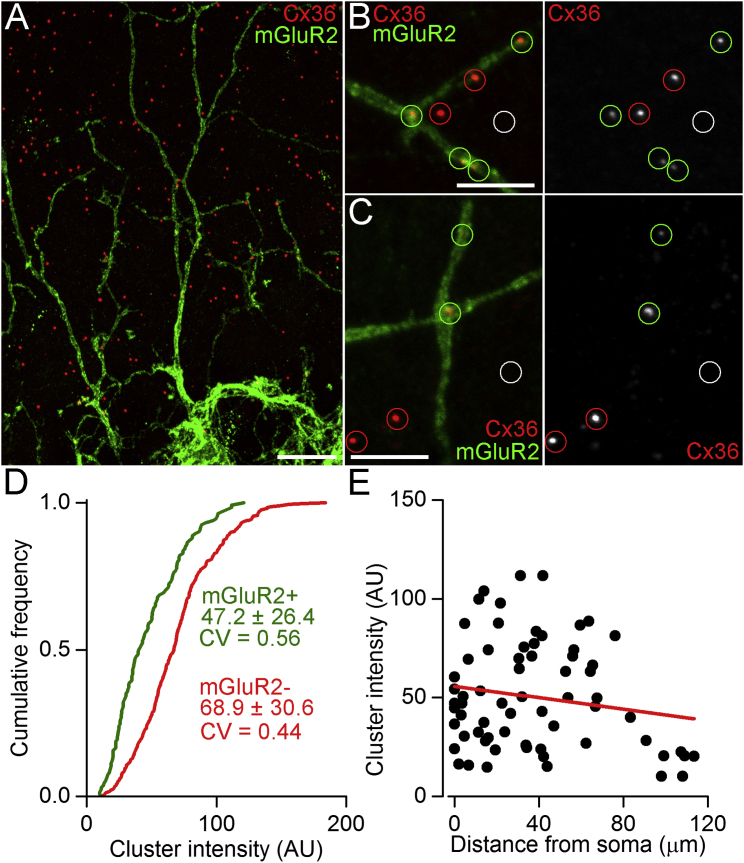
The Size of GJs Is Independent of Their Dendritic Location (A) Double immunofluorescent reaction for Cx36 (red) and mGluR2 (green) in the cerebellar ML. (B) High magnification confocal image showing only a fraction of the Cx36 puncta (green circles) are associated with mGluR2^+^ GoC dendrites. The majority of Cx36 immunopositive GJs (red circles) were present on putative stellate/basket or mGluR2^−^ GoCs. The size of Cx36 positive GJs was assayed by integrating the fluorescence in circular regions of interest (ROI) positioned over Cx36 immunopositive puncta in single confocal sections. The background fluorescence was obtained from neighboring immunonegative areas (e.g., white circular ROI) and was subtracted from Cx36 fluorescent integrals. (C) As for (B), but for another example. (D) Distributions of Cx36 fluorescent intensities reveal that GJs associated with mGluR2^+^ dendrites (green, n = 134) are 32% smaller (p < 0.01; Kolmogorov-Smirnov test [KS test]) than GJs on mGluR2^−^ processes (red, n = 380). (E) The intensity of Cx36 immunofluorescent puncta associated with mGluR2^+^ dendrites showed no significant correlation (n = 68, R = −0.17, p = 0.16, Pearson’s correlation) with their distance from the soma. The scale bars represent 10 μm in (A) and 5 μm in (B) and (C).

To investigate the relationship between the strength of GJs and their location on GoC dendrites, we quantitatively examined the immunoreactivity for Cx36, which mediates electrical coupling in GoCs (Vervaeke et al., 2010). GoCs were identified using mGluR2, a selective marker that labels the majority of GoCs (Ohishi et al., 1994, Simat et al., 2007). Cx36 immunopositive puncta were most abundant in the molecular layer (ML) and were also found at the intersection of mGluR2 positive (mGluR2^+^) GoC dendrites (Figures 2A–2C; 7% of all puncta in the ML). We assayed the strength of Cx36 containing GJs by measuring the integral of fluorescence of the Cx36 puncta in confocal sections (Figures 2B and 2C). The distribution of the fluorescent intensity values (Figure 2D) showed large variability irrespective of whether they were associated with mGluR2^+^ GoC dendrites (coefficient of variation [CV] = 0.56, n = 134) or not (CV = 0.44, n = 380). The mean intensity of Cx36 puncta on mGluR2^+^ dendrites was significantly lower (32%, p < 0.01; Kolmogorov-Smirnov test) than that on mGluR2 negative (mGluR2^−^) processes. Cx36 positive puncta in mGluR2^−^ processes are likely to belong to ML INs and mGluR2^−^ GoCs (Simat et al., 2007). To quantify how the intensity of Cx36 positive puncta depends on dendritic location, we acquired image stacks with a confocal microscope and performed partial 3D reconstructions of mGluR2^+^ GoCs within our 60 μm thick sections. We traced all Cx36 positive puncta on the reconstructed cells, allowing the simultaneous measurement of their fluorescent intensity and their distance from the GoC soma. The intensity distribution of Cx36 puncta located on mGluR2^+^ dendrites also exhibited a large variability (CV = 0.54, n = 68) and there was no correlation with the distance from the soma (Figure 2E; R = −0.17, p = 0.16, Pearson’s correlation). From the 3D reconstructions of mGluR2^+^ GoCs, the measured dendritic diameter showed a significant negative correlation with their distance from the soma (Figure S1A; R = −0.58, p < 0.01, Pearson’s correlation). We also determined the relationship between the Cx36 cluster intensity and the dendritic diameter and found no significant correlation (Figure S1B; R = −0.037, p = 0.78, Pearson’s correlation). These results suggest that GJs between GABAergic INs of the cerebellar cortex are highly variable in size and that the strength of GJs on GoCs is independent of their dendritic location and dendritic diameter.

### Passive Electrical Properties of GoCs

**Figure 3:**
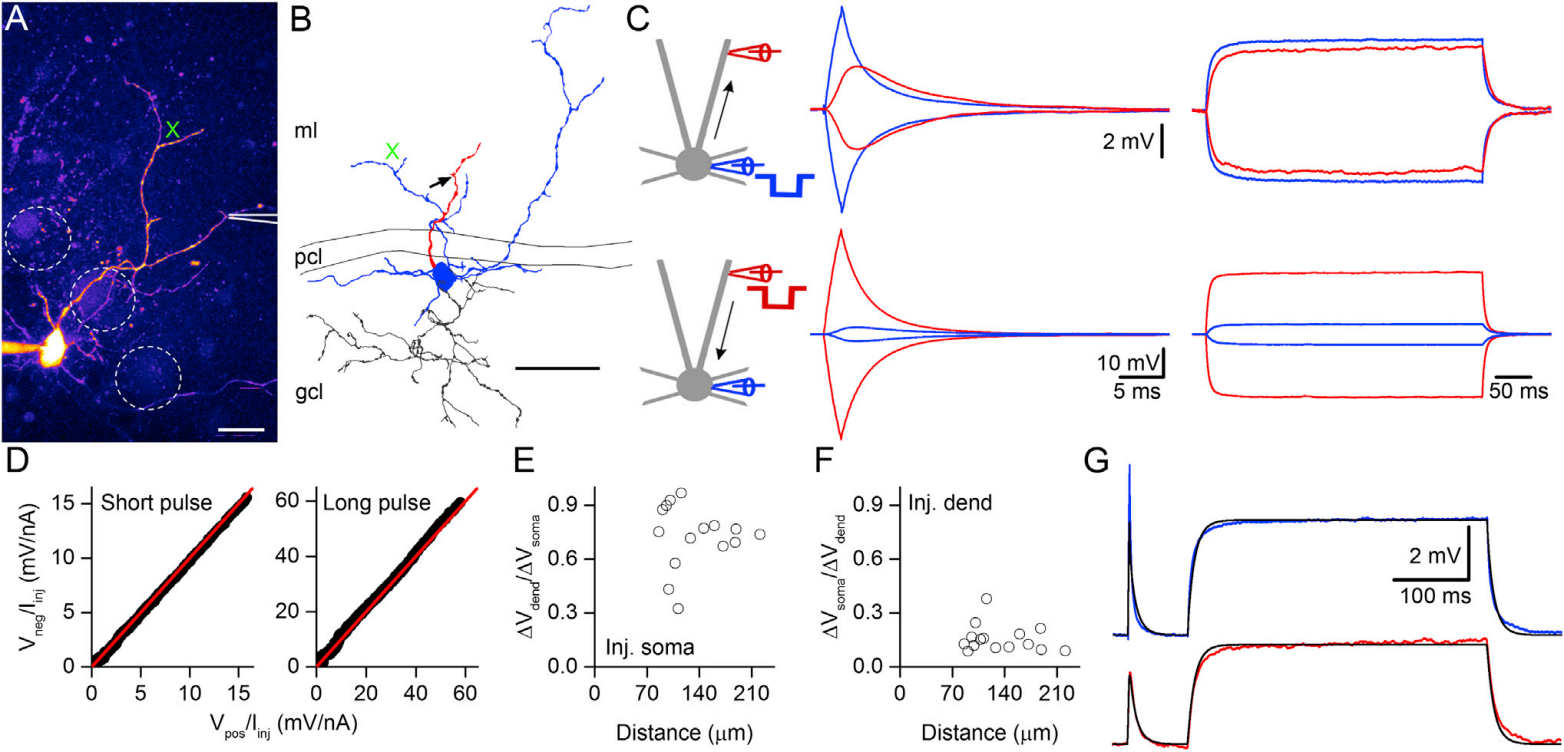
Characterization of Passive Voltage Propagation in GoC Dendrites (A) A two-photon maximum intensity projection image of an Alexa 594-filled GoC. A second pipette without Alexa 594 was used to simultaneously patch a dendrite (indicated by the cartoon). The dashed circles represent Purkinje cell somata. (B) Neurolucida reconstruction of the cell shown in (A) with dendrites colored in blue and the axon in gray. The patched dendrite is red. The arrow indicates the site of dendritic recording. The green cross indicates a neighboring dendritic branch and is present in both (A) and (B) for clarity. (C) Simultaneously recorded somatic (blue traces) and dendritic (red traces) voltage responses in the GoC shown in (A) evoked by somatic (top traces) and dendritic (bottom traces) short (left, ± 200 pA, 2 ms) and long (right, ± 50 pA, 400 ms) current injections in the presence of a cocktail of antagonists and channel blockers. (D) Linearity curves from the recorded voltage traces shown in (C), indicating no rectification of the somatic voltage responses to short (left) and long (right) somatic current injections. The slopes of the linear fits were 0.96 for short and 1.04 for long pulses (identity line: red). (E and F) Summary of all control somato-dendritic recordings showing the normalized changes in the voltage responses to somatic (E) and dendritic (F) current injections as a function of distance of the dendritic pipettes from the soma. (G) Simultaneously recorded somatic (top, blue) and dendritic (bottom, red) voltage traces in response to somatic current injections from the cell shown in (A)–(C). The multi-compartmental model of the reconstructed cell was fitted (black traces) to the experimental traces. The best fit was obtained with a R_m_ of 3.2 kΩ·cm^2^, a R_a_ of 188.9 Ω·cm, and a C_m_ of 3.3 μF/cm^2^ (Abbreviations: molecular layer, ml; Purkinje cell layer, pcl; and granule cell layer, gcl). The scale bars represent 20 μm in (A) and 50 μm in (B).

If GJ strength does not depend on the dendritic location, how does the dendritic location of GJs affect the GJPs recorded at the soma? Passive cable parameters, such as specific membrane resistivity (R_m_), specific axial resistivity (R_a_), and specific membrane capacitance (C_m_), together with the geometry of the cells shape the amplitude and waveform of chemical postsynaptic potentials (Spruston, 2008). To investigate the impact of dendritic attenuation on the CC, we examined the passive properties of GoC dendrites by performing two-photon targeted dual soma-dendritic patch-clamp recordings from 15 GoCs in the presence of the cocktail of antagonists and blockers (Figures 3A and 3B). Simultaneous somatic and dendritic voltage responses were recorded during short (2 ms, ± 200 pA) and long (400 ms, ± 50 pA) current injections (Figure 3C). The symmetry of the depolarizing and hyperpolarizing responses to either somatic or dendritic current injections indicated that the membrane was linear (Figure 3C). Moreover, the slopes of linear regression to short and long current injection-evoked voltage responses were close to one (0.96 and 1.04; Figure 3D). The steady-state dendritic attenuation was then quantified by plotting the relative changes in the voltage in the soma and dendrite as a function of distance. Dendrite to soma voltage attenuation was substantially larger than soma to dendrite attenuation as expected from the impedance mismatch between the large soma and fine dendrites (Figures 3E and 3F).

To determine the passive electrical properties of GoCs, we then carried out post hoc LM reconstructions of those cells where the morphology was sufficiently preserved (Figure 3B), built a multi-compartmental model of each cell in the NEURON modeling environment, and fitted the model to the electrophysiological data recorded from that cell. Since the electrical membrane properties of these GoCs were passive, we modeled the cells with a single leak conductance inserted into all compartments with a uniform density. By varying the values of R_m_, R_a_, and C_m_, we obtained a good fit to the recorded traces (Figure 3G), resulting in an R_m_ of 5.5 ± 2.3 kΩ·cm^2^, a R_a_ of 206 ± 81 Ω·cm, and a C_m_ of 2.7 ± 0.7 μF/cm^2^ (n = 5 cells). These values of R_a_ and C_m_ are considerably larger than those obtained from other neurons, suggesting that either GoC passive properties are different or that these properties are influenced by the electrically coupled syncytium within which the GoCs are embedded (Alcami and Marty, 2013). To distinguish between these possibilities, we performed direct measurements of GoC membrane properties and explored, with modeling, how electrical coupling could affect our estimates of passive properties.

### Determination of the Specific Membrane Capacitance of GoCs

**Figure 4:**
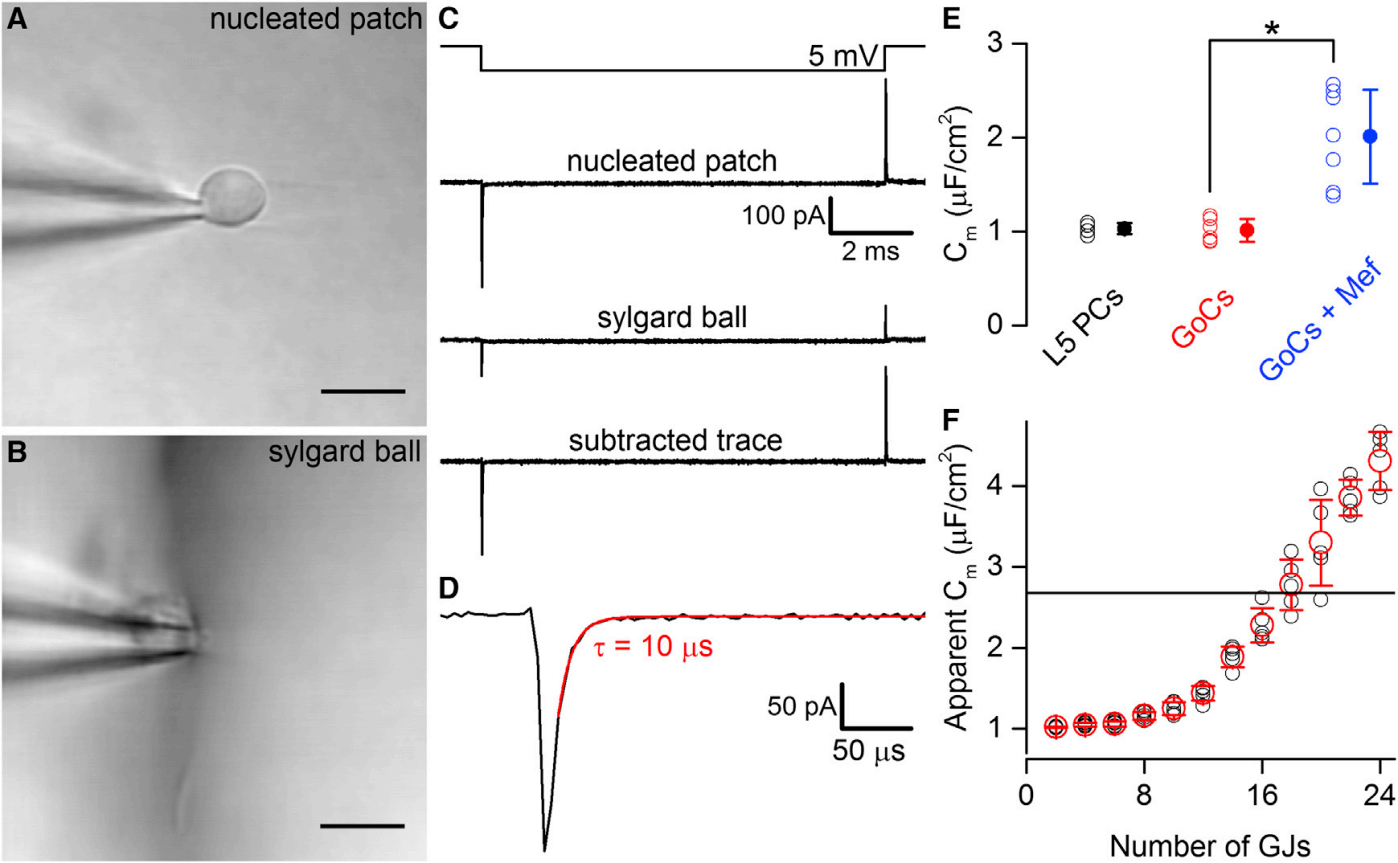
Measurement of the C_m_ of GoCs (A) A nucleated patch from a GoC visualized using Dodt contrast microscopy. (B) Image of the pipette pressed against a Sylgard ball creating a giga-seal. (C) Current transients recorded from the nucleated patch (top current trace) upon a −5 mV voltage command step. At the end of the recording, the residual capacitive transient was measured by pressing a Sylgard ball against the patch pipette (middle trace). The bottom image shows the subtracted capacitive transients. All of the traces are averages of 200 responses. (D) The capacitive current is shown on an expanded timescale. The decay of the capacitive transient was fitted with a single exponential function (red). (E) Summary graph of the C_m_ of layer 5 PCs (L5 PCs, n = 4), cerebellar GoCs in control condition (red, n = 6), and in 25 μM mefloquine (blue, n = 7). (F) Relationship between apparent C_m_ and number of GJs computed from GoC syncytium models. A central GoC was connected to increasing numbers of GoCs with two GJs per connected cells. When the central cell was connected to nine neighboring GoCs with 18 GJs, the apparent C_m_ was close to the single cell-approximated C_m_ of 2.7 μF/cm^2^. The black open circles represent individual syncytia and the red open circles represent mean ± SD. The scale bars represent 10 μm in (A) and (B).

To investigate the cause of the discrepancy between our C_m_ estimate and the generally accepted value of ∼1 μF/cm^2^, we measured the C_m_ experimentally. To test whether our protocol of measuring capacitance from nucleated patches (Figures 4A–4D) gave a reasonable estimate of C_m_, we first examined nucleated patches from neocortical layer 5 pyramidal cells (PCs). This gave a C_m_ value of 1.03 ± 0.06 μF/cm^2^ (n = 4; Figure 4E), similar to previously published values in this cell type (0.9 μF/cm^2^ in Gentet et al. [2000] and 1.2 μF/cm^2^ in Larkum et al. [2009]). Repeating these experiments in GoCs yielded a C_m_ of 1.01 ± 0.12 μF/cm^2^ (n = 6; Figure 4E). These results demonstrate that the membrane properties of GoCs are similar to those of other central neurons. To examine the effect of the syncytium on the “apparent C_m_”, we simulated networks with 1 to 12 cells coupled to a central GoC by two GJs, each having a conductance of 1 nS. We fixed the C_m_ to 1 μF/cm^2^ and the R_in_ to 120 MΩ in all GoCs based on the population average of our control cells (n = 15) and injected currents into the central neuron and recorded the voltage response. Next, we determined the apparent C_m_ of the central neuron following its disconnection from the syncytium (by setting the G_GJ_ to 0) by altering its C_m_ value until its voltage response matched that obtained when it was embedded within the syncytium. This apparent C_m_ increased monotonically as a function of GoC syncytium size and, when nine GoCs were connected to the “central” neuron via 18 GJs, the apparent C_m_ matched the value obtained from our experiments (Figure 4F). These results demonstrate that the large apparent C_m_ arises from the electrical coupling to the GoC syncytium and suggest that well established methods for estimating neuronal passive properties are not directly applicable to electrically coupled networks.

### Determination of the Specific Axial Resistance of GoC Dendrites

We next examined whether the presence of electrical synapses on GoC dendrites could also influence our estimate of R_a_. To do this, we generated ten electrically coupled GoC syncytia, each one modeled as a central cell with ten neighboring cells coupled by two GJs of 1 nS each, randomly placed over the dendritic tree. We then iterated R_m_, C_m_, and R_a_ to fit to the somatic and dendritic voltage responses evoked by a somatic current injection. Different GJ distributions resulted in a large variability in the estimated R_a_ values (Figure S2). Four GJs in the vicinity of the dendritic recording pipette resulted in the lowest R_a_ (62 Ω·cm; Figure S2B), whereas the highest R_a_ (318 Ω·cm) was obtained from a syncytium in which not a single GJ was present on the recorded dendrite (Figure S2C). These results demonstrate that the dendritic locations of GJs have a profound influence on the estimated R_a_.

**Figure 5:**
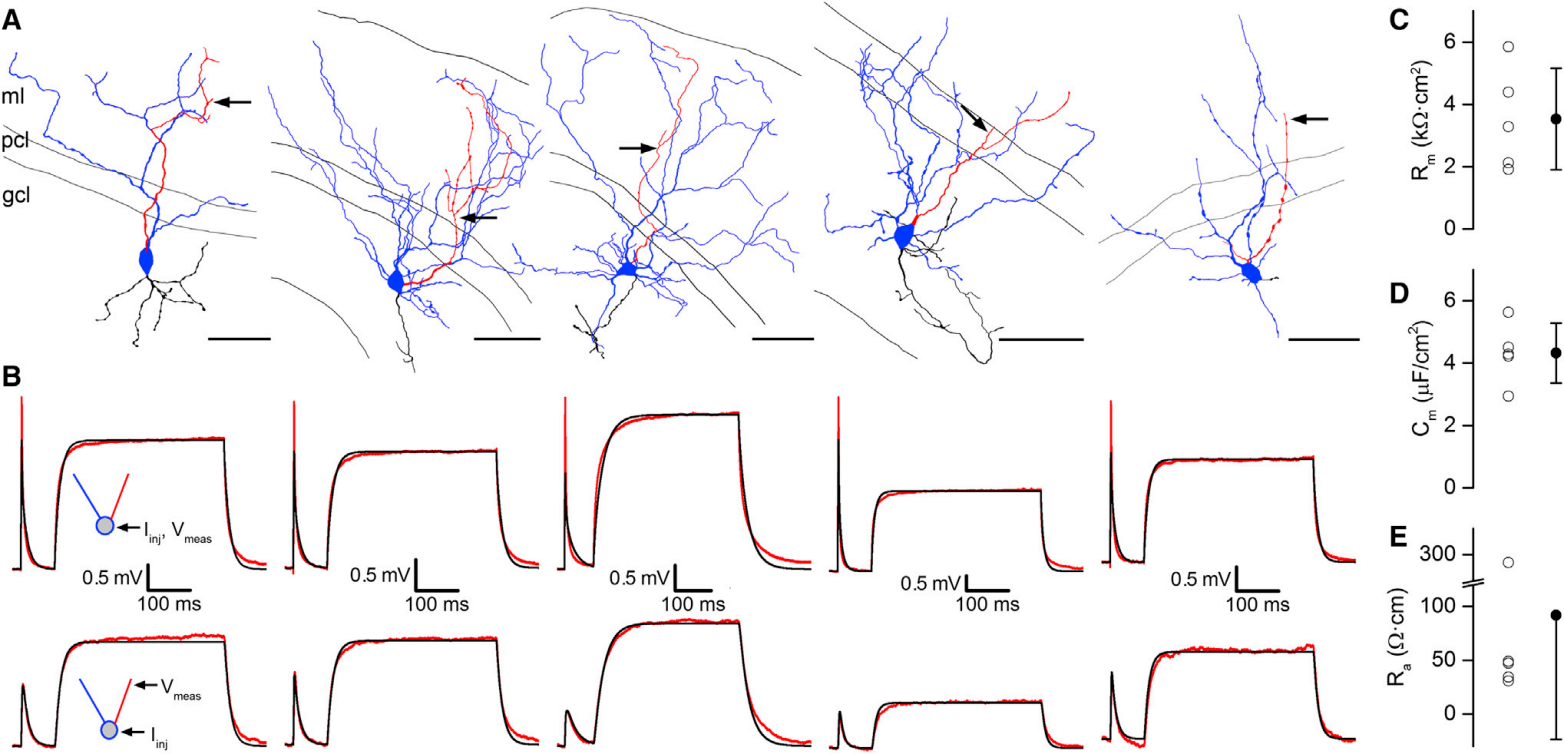
Estimation of the R_a_ of GoC Dendrites (A) LM reconstruction of five recorded and biocytin filled GoCs with partially reconstructed axons (blue: soma and dendrites; red: patch pipette targeted dendrites; and black: truncated axons). Dual somato-dendritic recordings were performed in the presence of a cocktail of antagonists and channel blockers plus 25 μM mefloquine and 10 μM 4-AP. The arrows indicate the position of the dendritic patch pipettes. (B) Somatic (top) and dendritic (bottom) voltage traces (red) in response to somatic current injections (100 pA, 2 ms and 20 pA, 400 ms) recorded from the cells shown in (A). R_m_, R_a_, and C_m_ were fitted (black). (C) Values of R_m_ obtained from fitting models of the 5 GoCs shown in (A) and (B). Filled symbols are means ± SD. (D) Values of C_m_ obtained from fitting models of the 5 GoCs shown in (A) and (B). Filled symbols and errors as for (C). (E) Values of R_a_ obtained from fitting models of the 5 GoCs shown in (A) and (B). Filled symbols and errors as for (C). The scale bars represent 50 μm in (A).

Because we could not determine the exact location and strength of each GJ on our recorded and reconstructed GoCs, we estimated the R_a_ following the pharmacological blockage of GJs with 25 μM mefloquine, a Cx36 channel blocker (Cruikshank et al., 2004). To ensure a sufficient level of block, we monitored the time-dependent effects of mefloquine by recording from three electrically coupled GoC pairs during wash in of the drug. In the presence of mefloquine, the R_in_ increased and the CC decreased during our recordings. Mefloquine increased R_in_ by 51% at the end of the 85 min wash in period (Figure S3A), consistent with the difference in R_in_ for wild-type and Cx36^−/−^ mice (Vervaeke et al., 2012) and decreased the CC by 74% (Figure S3B), which corresponded to a 81% ± 6% block of the Cx36 channels (Figure S3E). As stable dual somato-dendritic recordings cannot be maintained for such a long time, we preincubated our slices in 25 μM mefloquine and performed the recordings in steady-state conditions. This approach, which allows the comparison of populations of cells under control and mefloquine conditions, revealed that R_in_ of GoCs in the presence of mefloquine (182 ± 65 MΩ, n = 14) was 52% larger compared to that of control cells (120 ± 40 MΩ, n = 15, p < 0.05, unpaired t test). Five of the recorded cells were then post hoc reconstructed (Figure 5) and were used for modeling. In these models, the R_m_, R_a_, and C_m_ were iterated to obtain the best fit to the somatically and dendritically recorded traces upon somatic current injections (Figure 5B), resulting in an R_m_ of 3.5 ± 1.6 kΩ·cm^2^, a C_m_ of 4.3 ± 1 μF/cm^2^, and an R_a_ of 92 ± 115 Ω·cm (Figures 5C–5E). Interestingly, even though the majority of Cx36 channels were blocked by mefloquine, our C_m_ estimate remained high (Figure 5D). To investigate this unexpected result, we made recordings from nucleated patches pulled from GoCs preincubated in 25 μM mefloquine (Figure 4E). These experiments revealed that in the presence of mefloquine, the C_m_ was 2.01 ± 0.5 μF/cm^2^ (n = 7), significantly higher than in control (p = 0.002, unpaired t test). This suggests that mefloquine binds to the membrane and increases C_m_, consistent with a previous study, showing that mefloquine binds to membrane phospholipids (Chevli and Fitch, 1982).

### Estimation of GJ Plaque Conductance by Modeling GoC Pairs Embedded within a Syncytium

**Figure 6:**
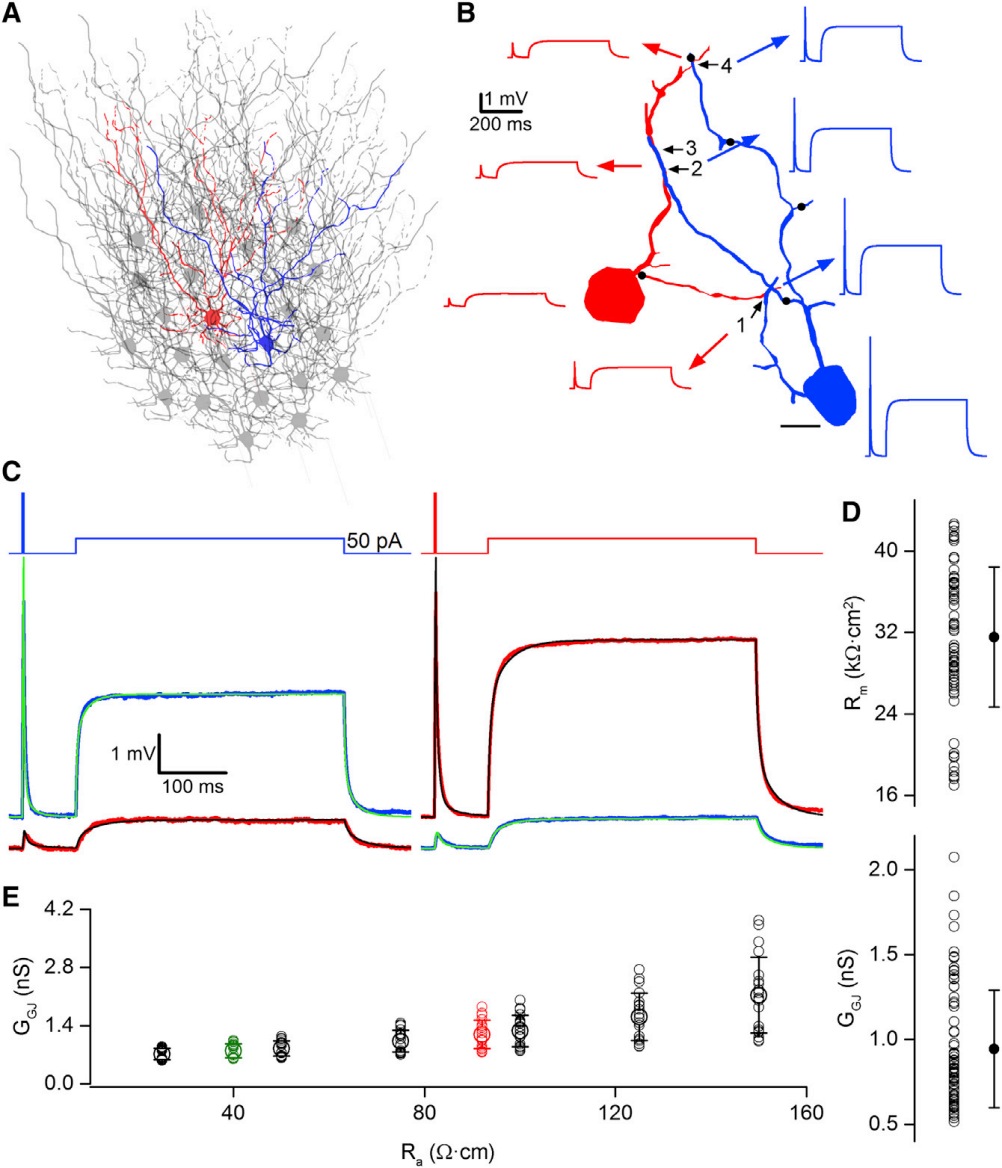
Determining G_GJ_ with Paired Recordings and Modeling of Electrically Coupled GoC Networks (A) Schematic of the modeled GoC syncytium in which the red and blue cells are embedded for determining the G_GJ_. (B) Attenuation of the voltage response from the somata of the blue to red cell along the dendrites and through the GJs obtained from the modeled GoC pair shown in Figure 1A. Each GoC was part of a syncytium (ten other GoCs were connected to the red and blue cell through 20 GJs). The locations of the randomly distributed GJs forming the syncytium are indicated by black dots on the perisomatic dendrites. (C) Voltage responses to short (200 pA, 2 ms) and long (50 pA, 400 ms) current injections in the connected cells shown in (B). R_m_ and G_GJ_ were iterated to obtain the best fit (black and green traces) to the recorded traces, while C_m_ and R_a_ were kept constant (1 μF/cm^2^ and 92 Ω·cm, respectively). (D) R_m_ (upper) and G_GJ_ (lower) values obtained from simulations. For each pair, ten randomly connected syncytia were created and R_m_ and G_GJ_ were determined from the red to the blue and from the blue to the red cell, resulting in a total of 80 R_m_ and G_GJ_ estimates (open circles). The filled symbols are means ± SD. (E) Dependence of G_GJ_ on R_a_. The small open circles indicate G_GJ_ of ten random syncytia. The large open circles are means ± SD. The red circles correspond to the mean R_a_ estimate of the five GoCs shown in Figure 5 (92 Ω·cm), and the green circles correspond to the average R_a_ estimate (40 Ω·cm) of four GoCs excluding the outlier cell in Figure 5E. The scale bar represents 10 μm in (B).

Classical methods of estimating G_GJ_ from experimentally measured R_in_ and CCs are based on single compartmental models connected by a resistor to represent the electrical synapse (Bennett, 1966, Devor and Yarom, 2002, Fortier and Bagna, 2006). This approach lumps together the voltage attenuation along the dendrites and through GJs into a single value. To directly address their individual contributions and to estimate G_GJ_, we constructed multi-compartmental models of the reconstructed cell pairs using the experimentally measured GJ locations (Figures 1 and 6), R_a_ (92 Ω·cm; Figure 5), and C_m_ (1 μF/cm^2^; Figure 4) and embedded them in two syncytia with each central cell being connected to ten other GoCs through 20 GJs randomly distributed on the dendritic tree (Figure 6A). In this way, we generated ten syncytia for each of our four reconstructed pairs. We then fitted the R_m_ in one of the cells to match its somatic voltage and the G_GJ_ to obtain the best fit to the membrane response in the connected cell (Figures 6B and 6C). The sequential fitting of R_m_ and G_GJ_ was iterated until their values changed by less than 5%. This approach resulted in a mean R_m_ of 32 ± 7 kΩ·cm^2^ (Figure 6D, upper) and a mean G_GJ_ of 0.94 ± 0.35 nS (Figure 6D, lower; n = 4 reconstructed pairs).

To determine the robustness of our estimate of G_GJ_, we tested the dependence of G_GJ_ on the R_a_ value using the fitting procedure described above (Figure 6E). These simulations showed that within a biologically plausible range of R_a_ values, G_GJ_ showed a systematic, but relatively small change. When we compared the G_GJ_ estimates obtained with an R_a_ of 92 Ω·cm (mean of the five soma-dendritically recorded GoCs; Figure 5E) versus 40 Ω·cm (average R_a_ value of these cells without one outlier cell), only a 34% reduction in the G_GJ_ was found (Figure 6E, red and green symbols).

### GJ Plaques Have Variable Sizes with a Constant Density of Connexons

**Figure 7:**
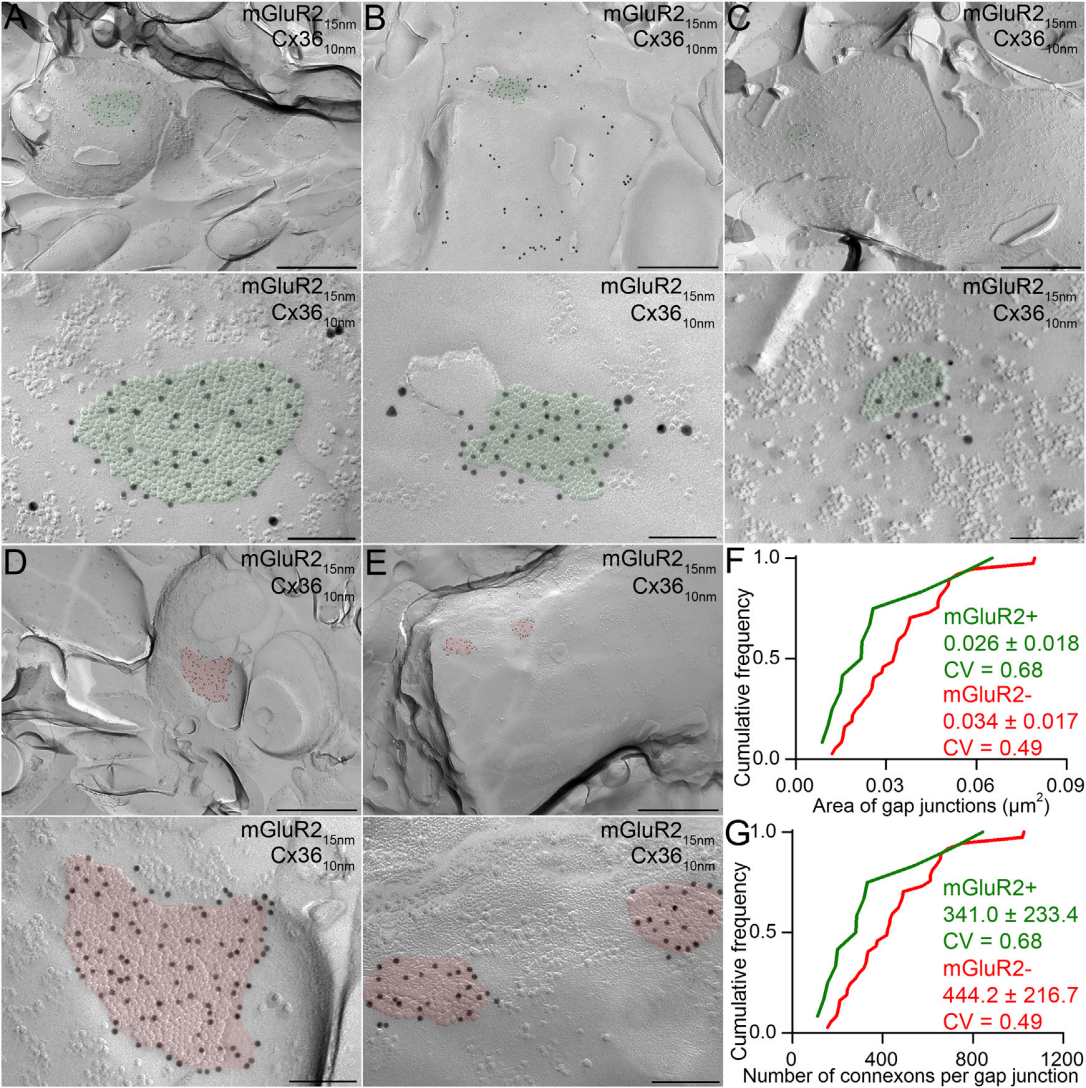
GJ Plaques have Variable Sizes, but a Constant Density of Connexons as Revealed by SDS-FRL (A) SDS-FRL of Cx36 (10 nm gold) and mGluR2 (15 nm gold) in the cerebellar cortex of young mice. Low (top panels) and high (bottom panels) magnification electron micrographs of GJs (highlighted in green) labeled for Cx36 on the P-face of mGluR2^+^ GoC dendrites reveal large heterogeneity in the area of GJ plaques. Gold particles labeling Cx36 are uniformly distributed over the GJ plaques characterized by the tight accumulation of small intra-membrane particles. (B) As for (A). (C) As for (A). (D) Low (top panels) and high (bottom panels) magnification electron micrographs showing GJs (highlighted by red) of variable sizes labeled for Cx36 on mGluR2^−^ dendrites in the ML. (E) As for (D). (F) Cumulative frequency distributions show heterogeneity in the GJ plaque area (mGluR2^+^ GJs: 0.009–0.065 μm^2^, n = 12 [green]; mGluR2^−^ GJs: 0.012–0.079 μm^2^, n = 37 [red]). GJ plaques on mGluR2^+^ GoCs were 24% smaller than those on mGluR2^−^ dendrites. (G) Cumulative probability plots reveal large heterogeneity in the number of connexons per GJ plaque (calculated from the area with an average connexon density of 12,940 ± 1,398/μm^2^). The scale bars represent 500 nm in (A)–(E), top, and 100 nm in (A)–(E), bottom.

Having quantified the mean conductance of GJ plaques, we next measured their size and the number of Cx36 channels (connexons) they contained by performing SDS-FRL immunogold double labeling for Cx36 and mGluR2 in the cerebellar cortex of young-adult mice. In the granule cell layer (GCL) many strongly mGluR2^+^ axonal membrane segments were found. Fewer gold particles were observed on somatic and dendritic plasma membranes of GoCs (Figure 7), consistent with the differences in the labeling intensities between GoC axons and dendrites observed in immunofluorescent reactions for mGluR2. In the ML, gold particles labeling Cx36 were only found in smooth, aspiny dendrites (Figures 7A–7E). Following the complete screening of 12 replicas of two mice, we found 12 GJ plaques (two in GCL and ten in ML) on mGluR2^+^ GoC dendrites. They were identified by the dense cluster of small intramembrane particles (IMPs) and by the accumulation of gold particles labeling Cx36 over the IMP clusters (Figures 7A–7C). The uniform distribution of Cx36 labeling and the fact that electrical coupling is absent in GoCs in Cx36^−/−^ mice (Vervaeke et al., 2010), argues against the potential presence of other types of connexin in these plaques (Li et al., 2008). It is therefore reasonable to assume that each IMP within the GJ plaque represents a connexon composed of six Cx36 subunits. The size of GJ plaques (0.026 ± 0.018 μm^2^) exhibited large variability on mGluR2^+^ GoC dendrites (CV = 0.68; Figure 7F), but the density of connexons per GJ plaques was rather uniform (12,940 ± 1,398 / μm^2^, CV = 0.11). Consequently, the number of connexons they contained (341 ± 233), calculated from the area and mean density of IMPs, also exhibited large variability. We also found GJ plaques homogeneously labeled for Cx36 in smooth mGluR2^−^ IN dendrites (Figures 7D and 7E) in the ML, but never observed them on spiny Purkinje cell dendrites. Large variability in the size (0.034 ± 0.017 μm^2^, CV = 0.49, n = 37 GJs) and connexon number (442 ± 217) was also found on mGluR2^−^ dendrites. Furthermore, GJ plaques on mGluR2^−^ dendrites were ∼30% larger than GJs on mGluR2^+^ dendrites (Figures 7F and 7G), consistent with the results of our immunofluorescent reactions (cf. Figures 7G and 2D). Calculation of the mean number of open Cx36 channels from the average conductance (0.94 nS) of a GJ plaque and the single channel conductance of a Cx36 channel (15 pS; Srinivas et al., 1999, Teubner et al., 2000) indicates that on average 63 of the 341 channels present within a GJ plaque are open and that the connexon open probability is 18%.

### Factors Contributing to the Variability in the Strength of Electrical Coupling between GoCs

**Figure 8:**
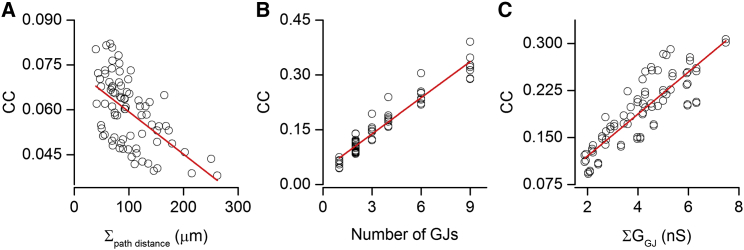
Contribution of Dendritic Location, Number, and Strength of GJs to the Variability in the Coupling Coefficient Estimated with Modeling of GoC Pairs (A) Relationship between coupling coefficient (CC) and the summed dendritic locations of the GJs. Red line is a linear regression fit (R = −0.57, p < 0.001, Pearson’s correlation). The mean CV of the CCs was 0.12 ± 0.03 (n = 8). (B) Relationship between CC and the number of GJs between two connected GoC. Red line is a linear regression fit (R = 0.96, p < 0.001, Pearson’s correlation). The mean CV of the CCs was 0.56 ± 0.03 (n = 8). (C) Relationship between CC and the sum of the conductance from 4 GJs with different strengths (determined from the measured distribution of GJ plaque area). Red line is a linear regression fit (R = 0.86, p < 0.001, Pearson’s correlation). The mean CV of the CCs was 0.24 ± 0.10 (n = 8).

Our results establish that the number (from one to nine, CV = 0.74; current data and that from Vervaeke et al., 2010) and size (from 0.009 to 0.065 μm^2^, CV = 0.68) of GJs, as well as their dendritic location (from 9 to 152 μm, CV = 0.57) are highly variable in electrically coupled GoCs. To estimate the relative contributions of these factors to the variability in the CC, we modeled GoC pairs, keeping the passive electrical properties constant (Figure 8). First, we randomly selected a distance from the EM identified GJ distances (51.3 ± 29 μm, n = 58 distances of 29 GJs in eight GoC pairs from this study and that of Vervaeke et al., 2010) and placed a GJ with a 1 nS conductance into a randomly selected dendrite of a GoC at this distance. The CC was then measured in both directions and the simulation was repeated ten times for each of the four GoC pairs, resulting in a mean CV of the CC of 0.12 ± 0.03 (n = 8; Figure 8A). Next, we tested the variability in CC due to different numbers of GJs between GoC pairs. The number of GJs between our EM analyzed eight GoC pairs ranged from one to nine, with a mean of 3.6 ± 2.7. In our simulations, we placed different numbers of GJs (all with a G_GJ_ of 1 nS) at ∼50 μm distance from the somata of both cells and calculated the CC for each pair. The mean CV of the CC was 0.56 ± 0.03 (n = 8; Figure 8B). Finally, we tested how the variability in G_GJ_ affects variations in the CC. Here, we placed four GJs (rounded from the mean GJ number of the eight EM analyzed GoC pairs) at ∼50 μm distances from the somata on randomly selected dendrites (one dendrite one GJ) and randomly selected the four G_GJ_ values from a population that was created from the distribution of GJ sizes. The mean GJ size distribution was normalized to the mean G_GJ_, resulting in a distribution of G_GJ_s that had a shape and variance of the distribution of the GJ areas with a mean of 0.94 nS. Ten repetitions of each simulation resulted in a mean CV of the CC of 0.24 ± 0.10 (n = 8; Figure 8C). Assuming that these three parameters are independent, the CV^2^ should add linearly, allowing us to account for the total variation in CC. Calculating the relative contributions of the CV^2^ due to the different dendritic locations of the GJs, different numbers of GJs, and different GJ sizes between the cells resulted in 4%, 81%, and 15% contributions, respectively.

## Discussion

We have investigated the functional properties of electrical synapses formed between the dendrites of inhibitory GoCs within the cerebellar cortex of young-adult mice. Our results demonstrate that the location of GJs has an impact on current flow within the dendritic tree, which affects the estimates of R_a_ and C_m_. By quantifying the dendritic properties under passive conditions, we have disentangled the contributions of the dendritic tree and the GJs to the coupling strength, allowing us to estimate the conductance of a dendritic GJ for the first time. Moreover, by quantifying the number of Cx36 channels per GJ plaque on GoC dendrites, we estimate that on average only 18% of the ∼340 channels are open. Our results also show that the number of GJ plaques is the major determinant of the strength and variability in electrical coupling between GoCs.

### Passive Membrane Properties of IN Dendrites and the Impact of GJs

In most INs, electrical synapses are located on thin dendrites, making the investigation of the mechanisms underlying the soma-to-soma voltage attenuation between two electrically coupled cells challenging. However, two-photon or confocal guided patch-clamp recordings from dendrites (Nevian et al., 2007) have allowed direct electrical access to thin IN dendrites, including those of basket cells of the hippocampus (Hu et al., 2010, Nörenberg et al., 2010) and GoCs of the cerebellum (Vervaeke et al., 2012). Here, we combined somato-dendritic paired whole-cell recordings from GoCs, post hoc reconstructions, and multi-compartmental modeling to quantify the passive electrical properties of fine (<1 μm) GoC dendrites and to determine their contribution to electrical signaling. In our initial experiments, which were carried out with GJ coupling intact, we obtained high values for R_a_ and C_m_. Our modeling of the GoC syncytium and subsequent direct experimental measurement of C_m_ in nucleated patches and R_a_ under conditions of GJ block showed that the presence of GJs distorted our estimates of both C_m_ and R_a_. While the larger apparent C_m_ can be understood intuitively due to the additional current required to charge up the membranes of the coupled cells (Alcami and Marty, 2013), the increase in apparent R_a_ was more difficult to understand because increasing the membrane conductance per se should not affect R_a_. However, simulations revealed that the specific location of individual GJs in relation to the dendritic recording site could have a profound effect on the R_a_ estimate. When many GJs are present on the recorded dendrite, more charge leaks out of the dendrite before reaching the soma than expected if the leak was uniformly distributed. In contrast, if no GJ was present on the recorded dendrite, less charge would leak out than expected. To fit the experimentally measured voltage attenuation with a model that assumes uniform R_m_, the R_a_ must be adjusted in opposite directions in these two cases, leading to highly variable estimates of R_a_. Indeed, the high impedance of fine IN dendrites makes them particularly sensitive to the presence or absence of a large G_GJ_. This, together with the relatively few GJs per cell (we estimate ∼20 for GoCs), may explain why R_a_ estimates from INs tend to be higher (e.g., 150 Ω·cm for cerebellar stellate cells, Abrahamsson et al. [2012], and 172 Ω·cm for dentate gyrus basket cells, Nörenberg et al. [2010]) than those from GJ-lacking principal cell dendrites (e.g., 115 Ω·cm for Purkinje cells, Roth and Häusser [2001], and 70 to 100 Ω·cm for layer 5 PCs, Stuart and Spruston [1998]; but see Golding et al. [2005], CA1 PC: 140–220 Ω·cm). To overcome the complicating effects of GJs, we performed experiments in the presence of a GJ blocker, resulting in a lower R_a_ estimate (92 Ω·cm). Our experimentally determined C_m_ value of 1 μF/cm^2^ is also similar to that obtained from other central neurons, suggesting that both R_a_ and C_m_ are relatively constant across central neurons. This contrasts with R_m_, which shows a large variation across cells with and without GJs (from 1 to >100 kΩ·cm^2^; Delvendahl et al., 2015, Major et al., 1994, Roth and Häusser, 2001).

### Mean G_GJ_, Number of Channels, and Open Probability

By quantifying the dendritic properties of GoCs under passive conditions and determining the number of GJs and their dendritic locations with LM and EM, we were able to account for the dendritic contribution to electrical coupling and quantify the mean conductance of dendritic GJs. The mean conductance of 0.94 nS is similar to the G_GJ_ estimates obtained for the large myelinated club endings of Mauthner cells (0.44 nS; Lin and Faber, 1988, Tuttle et al., 1986), but is considerably larger than that calculated for GJ plaques between mesencephalic trigeminal nucleus neurons (40–100 pS; Curti et al., 2012). Simply dividing the mean G_GJ_ by the single channel conductance of Cx36 channels (15 pS; Srinivas et al., 1999, Teubner et al., 2000) suggests that there are an average of 63 open channels per GJ. By applying a SDS-FRL immunolabeling approach, we were also able to determine the total number of channels per GJ from the clearly resolved IMPs. Gold particle labeling Cx36 was uniformly distributed on membrane surfaces that contained IMPs at a high density (13,000 per μm^2^; very similar to that reported by Kamasawa et al. [2006]). The lack of spacing between IMPs and the fact that electrical coupling is absent in Cx36^−/−^ mice indicate that each IMP within a GJ plaque represents a connexon channel and that the density of channels is close to the maximal packing density. Directly counting the IMPs within GJ plaques on GoCs suggests that there are on average 341 Cx36 channels per GJ. When combined with the number of open channels, this suggests that on average 18% of the Cx36 channels in a dendritic GJ are in an open state. In line with this estimate, a small fraction (15%–20%) of Cx35 (a fish ortholog of Cx36) channels are estimated to mediate electrical coupling in goldfish Mauthner cells (Lin and Faber, 1988, Pereda et al., 2003, Tuttle et al., 1986). In contrast, two recent studies reported much smaller (0.1% and 0.8%) estimates of the fraction of open Cx36 channels between the somata of neurons in the mesencephalic trigeminal nucleus (Curti et al., 2012) and in transfected HeLa cells and pancreatic β cells (Marandykina et al., 2013). However, these studies used diffraction-limited fluorescent microscopy for the measurements of GJ areas, which may have resulted in an overestimation of the GJ size and, as a consequence, an underestimation of the fraction of open channels. Nevertheless, a low open probability seems to be a general feature of GJs as the proportion of open Cx43, Cx45, and Cx57 channels are also low (Bukauskas et al., 2000, Palacios-Prado et al., 2009, Palacios-Prado et al., 2010). This could enable the strength of electrical coupling to be modulated rapidly without changing the number of channels in a plaque. Indeed, it has been demonstrated that n-alkanols and arachidonic acid (Marandykina et al., 2013), as well as changes in intracellular [Mg^2+^] (Palacios-Prado et al., 2013) can modify the number of functional Cx36 channels without altering their trafficking or single-channel conductance.

### Variation in GJ Size and Its Spatial Independence

Our immunofluorescent reactions and quantitative LM analysis suggest that GJs vary widely in strength, but do not vary systematically in size across the GoC dendritic tree. While it can be argued that the relationship between Cx36 immunofluorescent intensity and GJ strength is rather qualitative, the similar variation in GJ size in our SDS-FRL immunolabeling experiments, which unequivocally identified Cx36 immunopositive GJs in our replicas, supports the validity of this conclusion. Moreover, we show that the density of IMPs/Cx36 channels is relatively uniform, with small GJ to GJ variability (CV = 0.11), indicating a linear relationship between GJ size and channel number. In addition, when we compared the distributions of GJ areas on mGluR2^+^ and mGluR2^−^ dendrites in the ML, we observed a similar rightward shift on the cumulative probability plot to that of fluorescent intensities (cf. Figures 2D and 7F). These results confirmed that the relative fluorescent intensity of puncta provides a reliable estimate of the area and the total number of Cx36 channels. The high variability in the size and number of channels per GJ are properties that are also common to chemical synapses and may result from plasticity (Landisman and Connors, 2005, Pereda et al., 2013, Mathy et al., 2014).

Our results indicate that variation in the number of GJs between two connected GoCs (from 1 to 9; here and Vervaeke et al. [2010]) accounts for most of the variance in the CC. However, the availability of close dendritic appositions may limit the number of GJs that can be formed between two cells. As for chemical neurotransmission, the strength of electrical synapses could also be adjusted by changing the number of channels per synaptic contact. The 7-fold variability in the size of GJs (from 0.009 to 0.065 μm^2^) and, thus, the total number of Cx36 channels per GJ plaque, suggests that altering the number of channels per GJ could also be used as a way of changing G_GJ_. Both of these mechanisms would require insertion of new channels into the membrane. Consistent with these potential plasticity mechanisms, GJs are equipped with the machinery for fast changes, as the turnover of connexins in GJs can be quite rapid (1–3 hr; Beardslee et al., 1998, Gaietta et al., 2002) and interfering with Cx35 trafficking has been shown to modify the strength of electrical coupling in goldfish Mauthner cells (Flores et al., 2012).

### Origins of the Variability in Strength of Electrical Coupling and Its Relation to Synchrony

The large, slow inhibitory GJPs present in GoCs and the highly variable nature of the coupling strength enables appropriately timed excitatory chemical synaptic inputs to desynchronize the firing of the GoC network (Vervaeke et al., 2010). Our current results, which have quantified the relative contributions of dendritic attenuation, GJ size, and the number of GJs per electrical synaptic connections, indicate that the number of GJs per connection is the dominant determinant of the variation in CC, which is critical for spike time dispersion. Although our analysis was carried out under passive, steady-state conditions, it is likely to hold for the GJP under more physiological conditions, given their slow time course. Active conductances, such as the persistent Na^+^ conductance, which is located close to the soma in GoCs (Vervaeke et al., 2010), is also likely to modulate the amplitude of GJPs in a voltage-dependent manner (Dugué et al., 2009, Haas et al., 2011). However, the scaling is linear (Haas et al., 2011), so that at any given voltage, the variance of GJPs arising from different presynaptic cells is likely to be preserved. This high variability in electrical signaling, which is predominantly due to differences in the number of GJs, is likely to be advantageous for electrically coupled IN types that mediate desynchronization. However, this property of electrical synapses may be deleterious for neurons that utilize rapidly depolarizing spikelets to maintain spike synchrony under a range of conditions and those where electrical synapses mediate an early, precise readout of dendritic activation (Trenholm et al., 2014). In these cases, active dendritic conductances (Oesch et al., 2005, Sivyer and Williams, 2013) may not only counteract the low pass filtering of high-frequency components of spikelets, but it may also be important in normalizing the amplitude of GJPs, thereby counteracting variation in electrical coupling strength.

## Experimental Procedures

### Electrophysiology and Two-Photon Imaging

Sagittal slices (230 μm) of the cerebellar vermis were prepared from both male and female P23–P29 C57BL/6 mice in accordance with national and institutional guidelines, as described previously (Vervaeke et al., 2010; see Supplemental Information). Recordings were made at 32°C–36°C from cerebellar slices bathed in an artificial cerebral spinal fluid containing (mM): 0.001 TTX, 0.01 D-AP5, 0.01 NBQX, 0.01 SR95531, 0.0005 Strychnine, 0.01 ZD7288, and 0.1 Ba^2+^, and in a subset of experiments, 0.01 4-AP and 0.025 mefloquine. Data were recorded using the NeuroMatic software and analyzed using NeuroMatic and OriginPro (OriginLab). Membrane potentials are specified without correction for the liquid junction potential.

For two-photon targeted patching, GoCs were filled with 50 μM Alexa 594 (Invitrogen) through a somatic patch pipette. A second patch pipette without Alexa 594 and biocytin was used to patch one of the dendrites with the aid of an online overlay of the Dodt contrast and the fluorescence images (Nevian et al., 2007). Pipette capacitance neutralization and bridge-balance were applied and adjusted when necessary. Voltage signals were recorded using a MultiClamp 700B amplifier (Molecular Devices), low-pass filtered at 10 kHz, digitized at 20–40 kHz. Measurements of C_m_ were performed and analyzed as described previously (Gentet et al., 2000).

### Neurolucida Reconstructions and Multi-compartmental Modeling

Slices containing recorded cells were placed in a fixative containing 4% paraformaldehyde and 1.25% glutaraldehyde. Biocytin was visualized using avidin-biotin-horseradish peroxidase complex and a diaminobenzidine reaction. Sections were then dehydrated and embedded in epoxy resin (Durcupan). LM reconstructions of the cells were performed with the Neurolucida system (MicroBrightField). Light micrographs of each close apposition were used for guiding the EM identification of the GJs. All close appositions between the filled dendrites were checked in the EM (Tamás et al., 2000, Vervaeke et al., 2010). Multi-compartmental GoC models were constructed in either neuroConstruct (Gleeson et al., 2007) or NEURON (Carnevale and Hines, 2006) and simulations were performed with NEURON (version 7.3).

### Fluorescent Immunohistochemistry

A young (P26) male C57BL/6 mouse was anesthetized and perfused through the aorta with 3% paraformaldehyde and 0.2% picric acid in 0.1 M phosphate buffer (PB) for 20 min. Immunofluorescent reactions were carried out as described previously (Lorincz and Nusser, 2008; see Supplemental Information). The following primary antibodies were used: rabbit polyclonal anti-mGluR2/3 (1:500; Millipore Cat. No.: 06-676; RRID: AB_310212) and mouse monoclonal anti-Cx36 (1:1,000; Millipore Cat. No.: MAB3045; RRID: AB_94632). The specificity of the Cx36 immunolabeling under these experimental conditions was verified previously using Cx36^−/−^ mice (Vervaeke et al., 2010).

z stack images were acquired with a confocal microscope. Identical circular region of interests were positioned over Cx36 immunopositive puncta and the integral of Cx36 fluorescence was measured in the confocal section where the fluorescent intensity was the highest. mGluR2^+^ dendrites were reconstructed in 3D from confocal z stack images using the Neurolucida software and each Cx36 positive punctum was traced back to the parent soma to obtain the distance of Cx36 puncta from GoC somata.

### SDS-FRL

Two young (P22 and P26) male C57BL/6 mice were deeply anesthetized and were transcardially perfused with ice-cold fixative containing 2% paraformaldehyde in 0.1 M PB for 15 min. 80 μm thick sagittal sections from the cerebellar vermis were cut, cryoprotected in 30% glycerol, frozen with a high-pressure freezing machine (HPM100, Leica Microsystems), and fractured in a freeze-fracture machine (EM BAF060, Leica) as described in Lorincz and Nusser (2010) and the Supplemental Information.

The replicas were immunoreacted in the solution of rabbit polyclonal anti-mGluR2/3 (1:100; Millipore Cat. No.: 06-676; RRID: AB_310212 or Millipore Cat. No.: AB1553, RRID: AB_90767) and mouse monoclonal anti-Cx36 (1:500; Millipore Cat. No.: MAB3045; RRID: AB_94632) antibodies. This was followed by an incubation in the following secondary antibodies: goat anti-rabbit IgGs coupled to 15 nm gold particles and goat anti-mouse IgGs coupled to 10 nm gold particles (both 1:50; British Biocell). Finally, replicas were rinsed in TBS and distilled water before they were picked up on parallel bar copper grids and examined with a Jeol1011 EM (Jeol).

## Author Contributions

M.S. performed reconstructions and modeling; A.L. conducted reconstructions, correlated LM and EM and immunolocalizations; F.L. and K.V. performed the electrophysiological recordings and analysis; R.A.S. and Z.N. designed the study; and M.S., A.L., R.A.S., and Z.N. wrote the paper.

## Supplemental Information

Document S1. Supplemental Experimental Procedures and Figures S1–S3

Document S2. Article plus Supplemental Information
