# Supplementary material for: Functional Properties of Dendritic Gap Junctions in Cerebellar Golgi Cells
Source: Neuron. 2016 Jun 1;90(5):1043–56. doi: 10.1016/j.neuron.2016.03.029 (PMC4893164; doi:10.1016/j.neuron.2016.03.029)
Supplement: Document S1. Supplemental Experimental Procedures and Figures S1–S3 [file mmc1.pdf]

Neuron, Volume *90*

Supplemental Information

Functional Properties of Dendritic Gap Junctions in Cerebellar Golgi Cells

Miklos Szoboszlay, Andrea Lőrincz, Frederic Lanore, Koen Vervaeke, R. Angus Silver, and Zoltan Nusser

Supplemental Experimental Procedures:

Electrophysiology and two-photon imaging. Sagittal slices (230 µm) of the cerebellar vermis were prepared from both male and female P23 – P29 C57BL/6 mice in accordance with UK Home Office guidelines. Slices were prepared in a solution containing (in mM) 2.5 KCl, 4 $MgCl_2$, 0.5 $CaCl_2$, 1.25 NaH_2PO_4, 24 $NaHCO_3$, 25 glucose, 230 sucrose, bubbled with 95% O_2 and 5% CO_2. Recordings were made at 32-36 ºC from cerebellar slices perfused in ACSF containing (in mM) 125 NaCl, 2.5 KCl, 2 $CaCl_2$, 1 $MgCl_2$, 1.25 NaH_2PO_4, 26 $NaHCO_3$, and 25 glucose, 0.001 TTX, 0.01 D-AP5, 0.01 NBQX, 0.01 SR95531, 0.0005 Strychnine, 0.01 ZD7288, 0.1 Ba^{2+}, and in a subset of experiments, additional 0.01 4-AP and 0.025 mefloquine; pH = 7.3, equilibrated with 5% CO_2 and 95% O_2. Data were recorded using the Neuromatic software (www.neuromatic.thinkrandom.com, written in IGOR, Wavemetrics) and analyzed using Neuromatic and OriginPro (OriginLab). Membrane potentials are specified without correction for the liquid junction potential.

Two-photon imaging was performed with a microscope consisting of a Mai-Tai laser (Spectra-Physics, tuned to 880 nm), a galvanometer-based scanhead (Ultima, Prairie technologies) and an Olympus BX51 microscope with a 60x water immersion objective (NA = 1). For two-photon targeted patching, GoCs were filled with 50 µM Alexa594 (Invitrogen, Carlsbad, CA) through a somatic patch pipette containing (in mM) 120 K-gluconate, 20 KCl, 2 $MgCl_2$, 10 EGTA, 10 HEPES and 2 ATP-Na_2, titrated to pH = 7.3 with KOH, with 6 mM biocytin. A second patch pipette without Alexa594 and biocytin was used to patch one of the dendrites with the aid of an online overlay of the Dodt contrast and the fluorescence images (Nevian et al., 2007, Nat Neurosci, 10, 206). Pipettes were pulled from thick walled (outer diam.: 1.5 mm, inner diam.: 0.75 mm) borosilicate glass capillaries (Sutter Instruments) and had a resistance of 3 - 6 MΩ for somatic recordings and 9 - 20 MΩ for dendritic recordings. To minimize pipette capacitance, tips of the dendritic patch pipettes were coated with wax and the bath level was kept as low as possible. The series resistance (R_s) was 15 ± 5 MΩ for somatic and 67 ± 32 MΩ for dendritic recordings. Pipette capacitance compensation and bridge-balance were applied and adjusted when necessary during the experiments. The R_{in} at the soma and the steady-state voltage attenuation along the dendrites were measured by injecting 400 ms long hyperpolarizing current pulses of 50 pA (under control conditions) or 20 pA (in mefloquine). Voltage signals were recorded using a MultiClamp 700B amplifier (Molecular Devices), low-pass filtered at 10 kHz, digitized at 20 – 40 kHz.

Measurements of specific membrane capacitance (C_m). Pipette capacitance was compensated while in the cell-attached configuration on either cortical layer 5 pyramidal cells (n = 4) or in GoCs (n = 6 in control, n = 7 in 25 µM mefloquine). After establishing the whole-cell configuration with an internal solution containing (in mM) 125 Cs-gluconate, 2 $MgCl_2$, 10 EGTA, 10 HEPES, 20 KCl and 2 ATP-Na_2 titrated to pH 7.3 with CsOH, nucleated patches were pulled (Figure 4A). A -5 mV voltage command step was applied at a membrane potential of -60 mV for GoCs and -70 mV for pyramidal cells and 200 capacitive transients were recorded and averaged (main Figure 4C, D). Data were low-pass filtered at 49.9 kHz with an external 8-pole Bessel filter (Frequency Devices) and digitized at 200 kHz. At the end of the recording, the patch was ruptured and a sylgard ball was pressed against the tip of the pipette until a giga-seal formed (Figure 4B). The depth of the patch pipette in the bath solution was kept constant. The same pulse protocol was applied and the transient due to charging of the residual uncompensated pipette capacitance was recorded and averaged (Figure 4C). The residual capacitive transient was then

subtracted from the capacitive transient recorded from the nucleated patch. The capacitive transient was analyzed as previously described (Gentet et al., 2000, Biophys J, 79, 314). The amplitude, decay time constant (τ) and steady-state current transient (I_{ss}) were measured to estimate the series resistance of the recording pipette (R_s), the membrane resistance of the nucleated patch (R_p) and the membrane capacitance of the nucleated patch (C_p). R_s, R_p and C_p were determined using the following equations:

$$R_s = V_{step}/I_{peak(t=0)}$$

$$R_p = V_{step}/I_{ss} - R_s$$

$$C_p = \tau\ (1/R_s + 1/R_p)$$

Where V_{step} is the amplitude of the voltage step. The parameters τ and $I_{peak((t=0)}$ were determined by fitting a single exponential function to the averaged current transient (Figure 4D). The beginning of the fit was set 10 µs after the peak of the current transient. τ and the value of $I_{peak(t=0)}$ was determined by extrapolating the fitted exponential curve back to the start of the current response (t=0). The fit was rejected if the estimated value of R_s was less than the pipette resistance measured before the start of the recording. The data were also rejected if R_p was less than 500 MΩ. The value of C_p was divided by the measured surface area of the nucleated patch to calculate C_m.

It was assumed that the nucleated patches formed a prolate spheroid and the surface area was calculated using the following formula:

$$Surface\ area = 2\pi a^2 (1 + \frac{b}{ae} \sin^{-1} e)$$

Where a is the equatorial radius of the spheroid, and b is the distance from center to pole along the symmetry axis, where b > a and $e^2 = 1 - \frac{a^2}{b^2}$.

Computer simulations. GoCs were filled with biocytin during the electrophysiological experiments through the recording pipette, and visualized later by a DAB reaction for detailed morphological reconstruction using the Neurolucida software. GoC models were constructed in either neuroConstruct (Gleeson et al., 2007, Neuron, 54, 219) or NEURON (Carnevale and Hines, 2006, The NEURON Book, Cambridge Univ. Press) and simulations were run in NEURON (version 7.3).

To determine the specific axial resistance (R_a) of the cells, dual somato-dendritic recordings were performed from n = 29 cells (15 control and 14 in mefloquine), of which 5 recorded cells from each condition were reconstructed and their morphologies were imported into NEURON. Since these GoCs fulfilled our criteria of passiveness, only a leak conductance was inserted into all of the compartments with uniform density. Then the R_m, R_a and C_m parameters of the cells were iterated freely during the fitting procedure, to obtain the best fit to the somatic and dendritic current injection-evoked membrane voltage responses. Spatial discretization was applied as parameters changed according to the *d_lambda* rule, with a value of 0.1. This resulted in an average R_a value of 92 ± 115 Ω·cm for those GoCs recorded in mefloquine. We used the voltage responses generated by somatic current injections because the electrode series resistance was lower and pipette capacitance neutralization and bridge balance

compensation was less prone to error than for the dendritic recordings. However, somatic voltage responses to dendritic current injections were used to cross-check the parameters obtained from the best fit to somatic current injections.

To determine how the distribution of GJs influences the estimate of R_a, we modeled the GoCs in syncytia (Figure S2). The 'central' cell had 10 neighboring cells, each coupled by 2 GJs (resulted in n = 20 GJs) to the dedicated one. These GJs were randomly distributed on the dendritic tree of the neurons. We generated 10 of such random syncytia and then iterated the R_m, C_m and R_a parameters on the somatic current injection-evoked somatic and dendritic membrane voltage responses. During the simulation, the average conductance of GJs (G_{GJ}) was kept constant at 1 nS. The morphology of the cells was the same in each of the syncytia, as well as their passive electrical parameters.

To determine G_{GJ} based on our reconstructed GoC pairs (n = 4), we followed the same strategy described above by inserting a leak conductance with uniform density to all compartments of the neurons. Based on EM data, the exact locations of the GJs were set in the model with an accurate spatial discretization. First, we used the mean R_a value from our previous modeling and a C_m of 1 $\mu F/cm^2$, and then fitted the R_m parameter of one of the GoCs of a pair to obtain the best fit to its own somatic current-evoked voltage response. Parallel with this, we fitted G_{GJ} on the coupled cell's attenuated voltage response. Then we moved on to the other cell and repeated the same procedure. As the changes in R_m from the initial values also influenced G_{GJ}, we iterated this process until the change in R_m and G_{GJ} was less than 5%. We determined G_{GJ} in both directions (from cell blue to red and red to blue) for the 4 cell pairs in 10 random syncytia, resulting in an average G_{GJ} of 0.94 ± 0.35 nS, n = 80.

To explore the dependence of G_{GJ} on R_a as shown in Figure 6E, we followed the same fitting strategy described above fixing the R_a parameter of the cells at different values. The fitting of the model to the experimental data was done with NEURON's built-in Praxis fitting algorithm. Simulations were run on a desktop PC under Windows 7 using variable time step integration method "CVODE".

Simulations used to estimate the variability in CC arising from the dendritic location, GJ number and GJ strength were performed with simulations of a cell pair. Syncytia were not used because they would have introduced additional variation depending on the specific configuration. For these simulations an R_m value of 5 $k\Omega \cdot cm^2$ was chosen to match the average R_{in} of the modelled cells and the other passive properties were fixed at our measured values (R_a = 92 $\Omega \cdot cm$, C_m = 1 $\mu F/cm^2$). The CC was determined as the ratio of the post and presynaptic steady-state voltage responses upon long current injections.

Neurolucida reconstructions of GoCs and correlated EM. Slices containing recorded cells were placed in a fixative containing 4% paraformaldehyde and 1.25% glutaraldehyde in 0.1 M phosphate buffer (PB; pH=7.4). Slices were then cryoprotected in 10% and 20% sucrose solutions (in 0.1 M PB) for 45 min followed by rapid freezing and thawing in 0.1 M PB. After several washes in PB, slices were embedded in 1% agarose and re-sectioned at 60 µm thickness. Biocytin was visualized using avidin–biotin–horseradish peroxidase complex and a diaminobenzidine reaction. Sections were then dehydrated and embedded in epoxy resin (Durcupan). Three-dimensional LM reconstructions of the cells were performed with the Neurolucida system (MicroBrightField, Williston, VT) using a 100x oil-immersion objective (NA = 1.4). Light micrographs of each close apposition were used for guiding the EM identification of the GJs. Serial sections of 70 nm thickness were cut with an ultramicrotome. All close appositions

between the filled dendrites were checked in the EM (Tamas et al., 2000, Nat Neurosci, 3, 366; Vervaeke et al., 2010, Neuron, 67, 435).

Fluorescent immunohistochemistry. A young (P26) male C57BL/6 mouse was anesthetized and perfused through the aorta with 3% paraformaldehyde and 15 v/v% picric acid in 0.1 M PB (pH = 7.3) for 20 minutes. Immunofluorescent reactions were carried out as described previously (Lorincz and Nusser, 2008, J Neurosci, 28, 14329). Briefly, 60 μm thick sections from the cerebellar vermis were treated with 0.2 mg/ml pepsin in 0.2 M HCl at 37 °C for 10 minutes. Following a blocking step in 10% normal goat serum (NGS) made up in Tris-buffered saline (TBS, pH = 7.4), the sections were incubated in the mixture of rabbit polyclonal anti-mGluR2/3 (1:500; Millipore Cat. No.: 06-676; RRID: AB_310212) and mouse monoclonal anti-Cx36 (1:1000; Millipore Cat. No.: MAB3045; RRID: AB_94632) antibodies diluted in TBS containing 2% NGS and 0.1% Triton X-100. The following secondary antibodies were used to visualize the immunoreactions: Alexa488 conjugated goat anti-rabbit (1:500; Invitrogen) and Cy3 conjugated goat anti-mouse (1:500, Jackson ImmunoResearch, West Grove, PA) IgGs. The specificity of the Cx36 immunolabeling under these experimental conditions was verified previously using Cx36 knockout mice (Vervaeke et al., 2010, Neuron, 67, 435).

Z-stack images were collected (0.5 μm steps) with a confocal laser scanning microscope (FV1000, Olympus Europe, Hamburg, Germany) using a 60x (NA = 1.35) objective. Identical circular region of interests (ROI; area = 1.86 μm^2) were positioned over Cx36 immunopositive puncta and the integral of Cx36 fluorescence was measured. Most Cx36 clusters were present in three confocal sections represented by an intense spot in the central section. Measurements for each Cx36 punctum were performed in confocal sections in which the fluorescent intensity was the highest. Background fluorescence for the Cx36 immunosignal was measured in areas not containing Cx36 puncta (e.g. white ROI in Figure. 2B, C) and was subtracted from fluorescent integrals obtained for Cx36 puncta. Fluorescent integrals of Cx36 puncta and background did not change as a function of depth in the top 10 μm of the tissue, from where the data were collected. $mGluR2^+$ dendrites were reconstructed in 3D from confocal Z-stack images using the Neurolucida software and each Cx36 positive punctum was traced back to the parent soma to obtain the distance of Cx36 puncta from GoC somata.

SDS-digested freeze-fracture replica-labeling. Two young (P22 and P26) male C57BL/6 mice were deeply anesthetized and were transcardially perfused with ice-cold fixative containing 2% paraformaldehyde in 0.1 M PB for 15 minutes. 80 μm thick sagittal sections from the cerebellar vermis were cut, cryoprotected in 30% glycerol, frozen with a high-pressure freezing machine (HPM100, Leica Microsystems, Austria) and fractured in a freeze-fracture machine (EM BAF060, Leica) as described in Lorincz and Nusser (2010, Science, 328, 906). The fractured faces were coated on a rotating table by carbon (5 nm) with an electron beam gun positioned at 90°, then shadowed by platinum (2 nm) at 60° unidirectionally, followed by a final carbon coating (20 nm). Tissue debris were digested from the replicas with gentle stirring in a TBS solution containing 2.5% SDS and 20% sucrose (pH = 8.3) at 80°C for 18 hours. The replicas were then washed in TBS containing 0.05% bovine serum albumin (BSA) and blocked with 5% BSA in TBS for one hour followed by an incubation in the solution of rabbit polyclonal anti-mGluR2/3 (1:100; Millipore Cat. No.: 06-676; RRID: AB_310212 or Millipore Cat. No.: AB1553, RRID: AB_90767) and mouse monoclonal anti-Cx36 (1:500; Millipore Cat. No.: MAB3045; RRID: AB_94632) antibodies. This was followed by an incubation in 5% BSA in TBS containing the following

secondary antibodies: goat anti-rabbit IgGs coupled to 15 nm gold particles (1:50; British Biocell International, Cardiff, UK) and goat anti-mouse IgGs coupled to 10 nm gold particles (1:50; British Biocell). Finally, replicas were rinsed in TBS and distilled water, before they were picked up on parallel bar copper grids and examined with a Jeol1011 EM (Jeol, Tokyo, Japan).

Supplemental Figures:

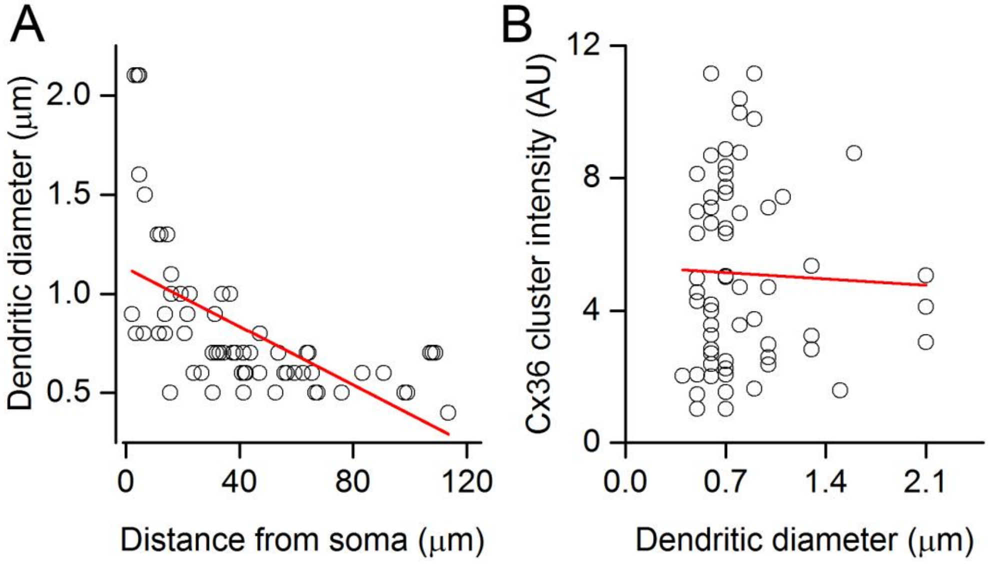

Figure S1 related to Figure 2. The intensity of Cx36 immunopositive clusters does not correlate with the dendritic diameter.

(A) Relationship between dendritic diameter and distance from the soma. Red line is a linear regression fit ($R = -0.58$, $p < 0.01$, Pearson's correlation).

(B) Relationship between intensity of Cx36 immunopositive clusters and dendritic diameter. Red line is a linear regression fit ($R = -0.037$, $p = 0.78$, Pearson's correlation).

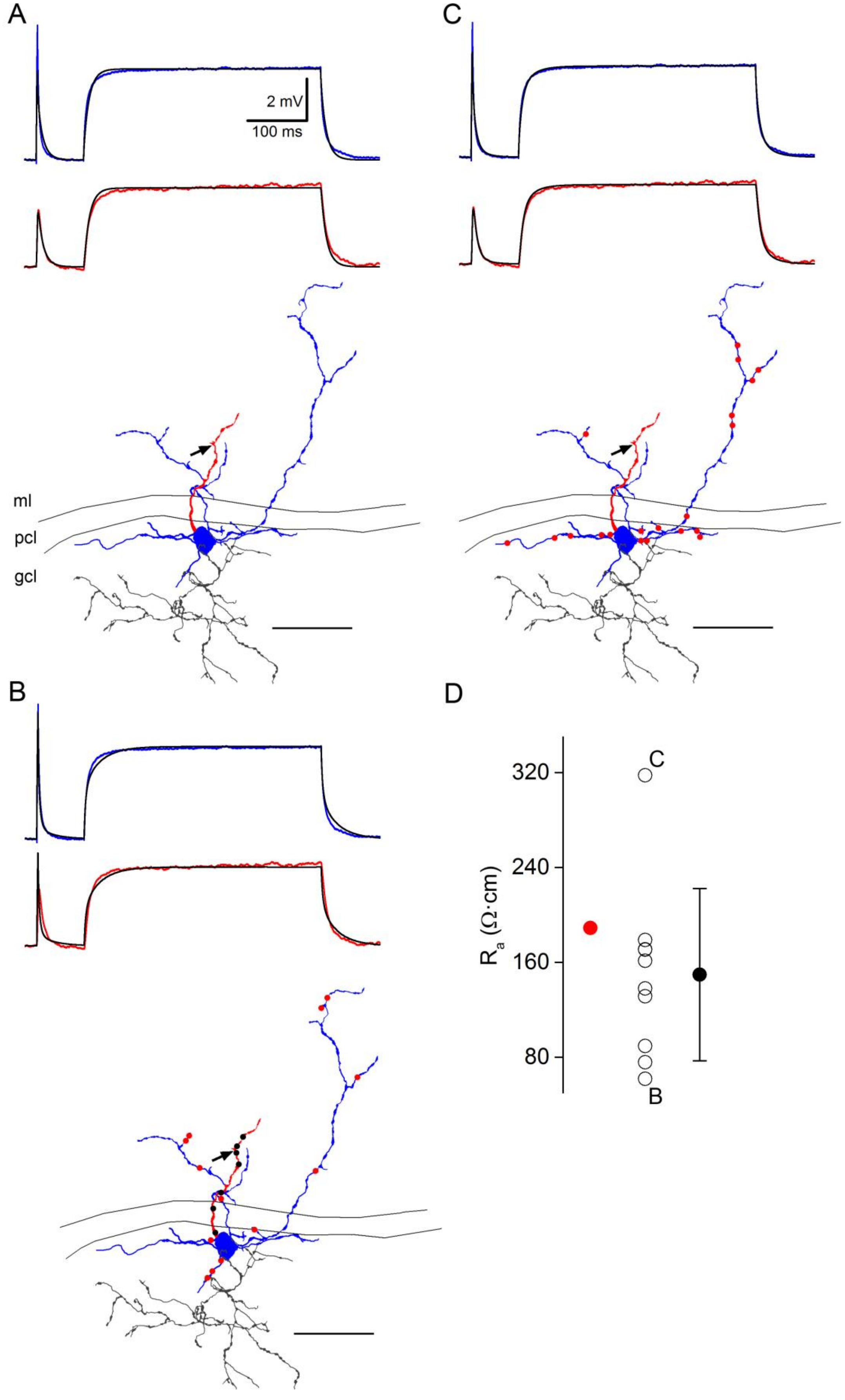
A
C
2 mV
100 ms
ml
pcl
gcl
B
D
R_a (Ω·cm)
320
240
160
80
C
B

Figure S2 related to Figure 3. Dependence of the apparent specific axial resistance on the exact locations of GJs

(A) Top: Simultaneously recorded somatic (blue traces) and dendritic (red traces) voltage traces in response to somatic current injections from the cell shown below. Multi-compartmental model of the reconstructed cell was fitted (black traces) to the experimental voltage traces. The best fit was obtained with a specific membrane resistance (R_m) of 3.2 kΩ·cm^2, a specific axial resistance (R_a) of 188.9 Ω·cm and a specific membrane capacitance (C_m) of 3.3 µF/cm^2. Bottom: Neurolucida reconstruction of the GoC. The dendrite targeted by the dendritic patch pipette is shown in red, the other dendrites and the soma are in blue, the truncated axon is in gray. The arrow indicates the location of the dendritic patch pipette.

(B, C) Top traces are the best fits obtained with 2 syncytia (out of 10 random syncytia) that produced the lowest (B) and highest (C) R_a estimates. GJ conductance was kept constant at 1 nS in all simulations (20 GJs, connecting 10 other cells). Dots indicate the locations of GJs on the dendritic tree (black dots on the red dendrite targeted by the dendritic patch pipette (arrow), red dots on the remaining part of the dendritic tree shown in blue).

(D) The estimated R_a from the 10 syncytia (black open circles are individual syncytia; filled circle is mean ± SD) revealed that randomly distributed gap junctions on the dendritic tree of GoCs strongly influence the estimated R_a. The R_a estimate of the single cell fitting (A) is shown in red.

Scale bars: 50 µm in (A-C)

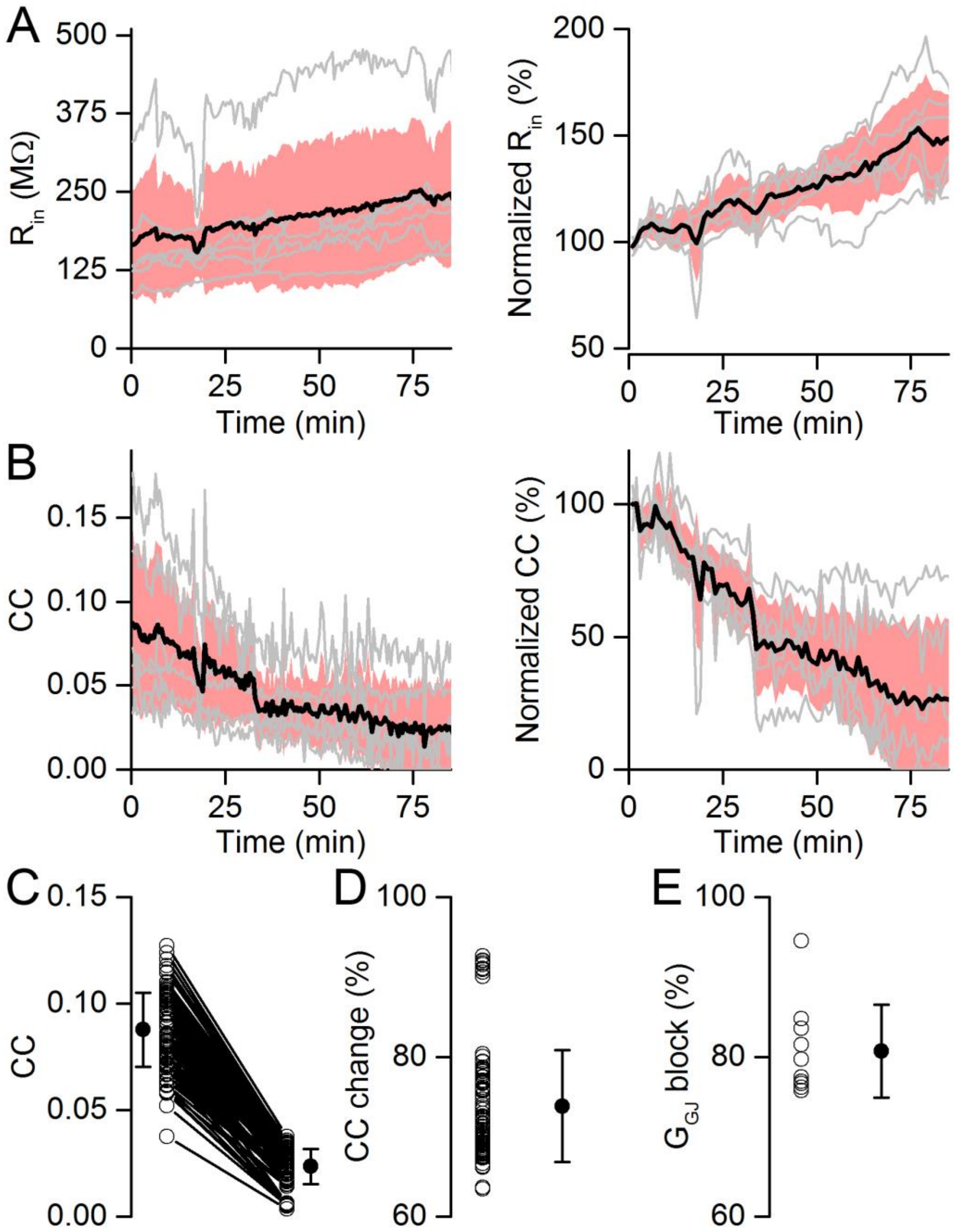

Figure S3 related to Figure 5. Effect of mefloquine on electrically coupled Golgi cells (GoCs).

(A) Absolute (left) and normalized (right) changes in the R_{in} (end: 51%) of electrically coupled GoCs (n = 3 GoC pairs) during the wash-in of 25 µM mefloquine.

(B) Absolute (left) and normalized (right) changes in the coupling coefficients (CC, n = 3 GoC pairs). The increase in R_{in} of the coupled cells is paralleled by a decrease in their CC during the wash-in of mefloquine, indicative of blocking GJs (absolute change in CC: from 0.087 ± 0.055 to 0.024 ± 0.031, relative change in CC: 74 ± 30%).

(C, D) Simulation of GoC syncytium (n = 10 syncytia, each with n = 10 coupled cells to the 'central' cell) reproduced the experimentally determined absolute (C, from 0.088 ± 0.018 to 0.024 ± 0.008) and relative (D, 74 ± 7%) changes in CC. The R_{in} change was constrained to be 52% (from population data) uniformly in all of the models when blocking GJs.

(E) The modelled syncytia of GoCs predict 81 ± 6% block of GJs by mefloquine. Black symbols are means ± SD.
